# Prophylaxis of Tetanus Resulting from Trench Feet

**Published:** 1918-01

**Authors:** 


***Prophylaxis of Tetanus Resulting from Trench Feet.*** By M. J. Bacri, Médecin-Major de 2e classe, and chief of an Ambulance. Trans. and Abs. from the *Archives de Médecine et de Pharmacie Militaires,* Oct., 1916, p. 470.

During two months in the winter of 1915 the author observed 4 cases of tetanus which developed among 25 cases of trench feet. The tetanus set in from 11 to 12 days after removal from the trenches. In only 1 of the 4 cases was there any indication of gangrene. In the other 3 there were merely the usual signs of freezing without the least break in the skin.

There can be little doubt that in all four cases tetanus arose from microscopic injuries. In case of a missile wound such men would have been treated preventively against tetanus. The author believes that, although tetanus of this kind is not frequent, there is always grave danger of its developing in light cases of trench feet. He therefore urges that all cases of trench feet be treated preventively for tetanus. (*See Medical Bulletin*, No. 1, for Nov. 1917, p. 35.)

